# Supplementary material for: High-intensity irreversible electroporation targeting intracellular structures enhance tumor ablation in lung cancer models
Source: PLoS One. 2026 Apr 22;21(4):e0346472. doi: 10.1371/journal.pone.0346472 (PMC13102207; doi:10.1371/journal.pone.0346472)
Supplement: Attachment — Submitted filename: PLOS ONE REVIEW_PS.docx [file pone.0346472.s001.docx]

**Comments to the Author:**

The manuscript entitled “High-intensity irreversible electroporation targeting intracellulafigr structures enhances tumor ablation in lung cancer models” presents an innovative and timely study investigating the effects of high-intensity irreversible electroporation (IRE) on intracellular structures, particularly nuclear membranes and mitochondria, in lung cancer models. The work combines simulation, in vitro experiments, and preliminary in vivo validation, offering significant insights into potential improvements in IRE-based tumor ablation.

Strengths:

- The study addresses a critical limitation of conventional IRE, i.e., incomplete intracellular damage leading to possible tumor recurrence.
- The combination of computational modeling, in vitro assays, and in vivo ultrastructural analysis provides a comprehensive assessment of high-field effects.
- The results are compelling, demonstrating that high electric field intensities induce significant mitochondrial dysfunction, oxidative stress, and nuclear membrane deformation, which could potentially improve tumor eradication.
- The manuscript is generally well organized and written in clear English.

**Areas for Improvement:**

1. Title Typographical Error:
   - The title contains a typographical error:

“intracellulafigr” → should be “intracellular”

2. Clarification of Energy Equivalence:
   - The manuscript frequently compares low electric field (LEF) and high electric field (HEF) conditions, stating that they delivered the “same total energy.” However, it is not entirely clear how this equivalence was calculated (e.g., purely Joule heating, energy per pulse, pulse shape effects). I recommend including a table summarizing pulse conditions and explicit calculations of delivered energy.
3. Statistical Details:
   - Although statistical analyses are reported, several figures lack precise p-values, and it is unclear which post hoc tests were applied after ANOVA. Please clarify statistical methods and ensure consistency across all figures.
4. In Vivo Data Expansion:
   - The in vivo data are limited to TEM analysis. It would significantly strengthen the manuscript to include:
     - Tumor volume measurements or necrosis quantification post-treatment
     - Histological analyses beyond TEM (e.g., H&E staining)
     - Data on potential side effects or systemic responses (e.g., inflammation, weight loss)
5. Thermal Considerations:
   - The discussion briefly mentions temperature increases during high-field IRE. Given that high electric fields might cause localized heating in tissues, additional discussion or in vivo measurements of thermal effects would be valuable for assessing clinical safety.
6. Terminology Consistency:
   - The manuscript switches between terms like HEF, high-voltage IRE, and high electric field. For clarity, please adopt consistent terminology throughout.
7. Language and Minor Errors:
   - Besides the title typo, minor wording issues are present, e.g.:
     - “The viabilities of 4000 and 5000 V/cm were almost similar each other…” → should read: “The viabilities at 4000 and 5000 V/cm were similar to each other.”
     - In several places, “Data is represented…” should read: “Data are represented…”
   - Please proofread the manuscript carefully for minor grammatical errors and ensure consistent spelling of technical terms (e.g., spell out “NO” as nitric oxide on first mention).

**Questions for the Authors:**

- Could you clarify precisely how “same total energy” was calculated for LEF and HEF conditions?
- Did you measure tissue temperatures in vivo to confirm that high-field protocols remain non-thermal in tissue?
- Were any physiological responses (e.g., muscle contractions) observed during in vivo HEF pulsing?
- Why were in vivo analyses limited to TEM without quantifying macroscopic tumor response?
- The data suggest suppressed transcriptional activity under HEF (e.g., low p53, ATR). Does this indicate necrosis as the dominant cell death pathway, and were any necrosis-specific markers assessed?
- Have you considered potential systemic toxicity from high oxidative stress observed in HEF-treated cells?
- Did you assess potential effects on normal lung tissue adjacent to treated tumors?
- How does your proposed HEF protocol compare to existing high-frequency IRE (HFIRE) approaches in terms of efficacy and safety?
- **Was current measured during the electroporation procedures, particularly in vivo, to monitor tissue conductivity changes or electrical dose delivered? If so, could you report those results?**

Overall, this is an interesting and promising manuscript addressing important challenges in cancer ablation therapy. The study would benefit from clarifying methodological details, expanding in vivo analyses, and addressing minor language issues. I look forward to the authors’ revisions.
