# Supplementary material for: High-intensity irreversible electroporation targeting intracellular structures enhance tumor ablation in lung cancer models
Source: PLoS One. 2026 Apr 22;21(4):e0346472. doi: 10.1371/journal.pone.0346472 (PMC13102207; doi:10.1371/journal.pone.0346472)
Supplement: Attachment — Submitted filename: Response to Reviewers(Jan. 12).docx [file pone.0346472.s004.docx]

Jan 12, 2026

Academic Editor, PLOS ONE,

Resubmission of Manuscript PONE-D-25-30916R1 (Correction of PDF Truncation Error)

Dear Dr. Planalp,

Thank you for returning our manuscript and bringing the issue of missing responses to our attention.

**Correction of PDF Submission Error**: Upon receiving your decision letter, we investigated our previous submission records and discovered that a PDF conversion error occurred during the system's file generation process, which truncated the end of our "Response to Reviewers" file. This technical glitch resulted in the unintentional omission of our responses to Reviewer 2 (Points 11–14).

We sincerely apologize for not making this technical error during the proofing stage. We have now re-uploaded the full "Response to Reviewers" document and have explicitly verified that all responses—including the detailed answers to Points 11–14 regarding resistance interpretation, gene expression controls, and citations—are fully visible in the generated PDF.

**Significance of the Work:** On behalf of my co-authors, I am pleased to resubmit the revised manuscript entitled “High-intensity irreversible electroporation targeting intracellular structures enhances tumor ablation in lung cancer models”.

This study provides new insight into how high electric-field irreversible electroporation (HEF-IRE), using short microsecond pulses, can reliably permeabilize the nuclear membrane and induce comprehensive intracellular damage—overcoming key limitations of standard IRE protocols. Our findings contribute mechanistic understanding that may guide the development of next-generation IRE strategies for improved oncologic outcomes.

We believe that this revised manuscript, with the complete response to all reviewer comments, now fully meets the high scientific and editorial standards of PLOS ONE.

Thank you for your time and consideration.

Sincerely,

Sung Bo Sim, M.D., Ph.D.

Department of Thoracic and Cardiovascular Surgery,

The Catholic University of Korea, Bucheon-si, 14647

Republic of Korea

Tel: +82-32-340-7143,

Fax: +82-32-340-7227,

E-mail: sungbo@catholic.ac.kr

============================================

Reviewer 1

============================================

1. Title Typographical Error:

The title contains a typographical error:

“intracellulafigr” → should be “intracellular”

Author Response:

We revised the title correctly.

Revision:

High-intensity irreversible electroporation targeting intracellular structures enhances tumor ablation in lung cancer models

2. Clarification of Energy Equivalence:

The manuscript frequently compares low electric field (LEF) and high electric field (HEF) conditions, stating that they delivered the “same total energy.” However, it is not entirely clear how this equivalence was calculated (e.g., purely Joule heating, energy per pulse, pulse shape effects). I recommend including a table summarizing pulse conditions and explicit calculations of delivered energy.

Author Response:

We appreciate your insightful comment. To clarify how energy equivalence between the LEF and HEF conditions was established, we have now added a detailed description and a new table summarizing the pulse parameters and corresponding energy indices.

Specifically, the “energy index” (V²·t·N) was used to represent the relative total electrical energy delivered per treatment, where V is the electric field strength (V/cm), t is the pulse width (s), and N is the number of pulses. This index reflects the proportional relationship to Joule energy (E ∝ V²·t·N/R), assuming a constant impedance across conditions.

The new table (Table 1) presents the calculated energy indices for all pulse settings applied at 1 Hz under both HEF and LEF protocols. This addition improves transparency and allows direct comparison of the total energy delivered under different electric field conditions.

| Voltage (V/cm) | | Pulse width (µs) | Energy index V^2^·t·N (V^2^·sec/cm^2^) | | |
| --- | --- | --- | --- | --- | --- |
|  |  |  | Pulse number | | |
|  |  |  | 10 | 20 | 40 |
| HEF | 1000 | 10 | 100 | 200 | 400 |
|  | 2000 | 10 | 400 | 800 | 1600 |
|  | 3000 | 10 | 900 | 1800 | 3600 |
|  | 4000 | 10 | 1600 | 3200 | 6400 |
|  |  | 20 | 0 | 6400 | 0 |
|  |  | 40 | 0 | 12800 | 0 |
|  | 5000 | 10 | 2500 | 5000 | 10000 |
|  | 6000 | 10 | 3600 | 0 | 0 |
| LEF |  | | Pulse number | | |
|  |  |  | 64 | | |
|  | 1000 | 100 | 6400 | | |
| Applied all to be frequency 1Hz | | | | | |

3. Statistical Details:

Although statistical analyses are reported, several figures lack precise p-values, and it is unclear which post hoc tests were applied after ANOVA. Please clarify statistical methods and ensure consistency across all figures.

Author Response:

Thank you for this valuable comment. In response, we thoroughly revised our statistical reporting for improved clarity and consistency. Specifically:

- All figures (Fig. 2–Fig. 6) were updated so that ANOVA-based comparisons are clearly indicated using the symbols #, ##, and ###, while t-test–based comparisons are marked using *, **, and ***.
- All figure legends were rewritten to explicitly state the statistical method used (ANOVA with Tukey’s HSD post hoc test or unpaired t-test), along with the meaning of each symbol and the sample size (n).
- The Statistical Analysis section in the Materials and Methods has been revised to clearly describe:
  - the use of one-way ANOVA followed by Tukey’s HSD post hoc test for multiple-group comparisons,
  - the use of unpaired two-tailed t-tests for two-group comparisons, and
  - the symbol system applied in the updated figures.
- Exact p-values, or t statistics, and degrees of freedom are now included in Supplementary Table S1 for full transparency.

Revision:

Figure:

Figure 2.

Figure 3.

Figure 4.

Figure 5.

Figure 6.

Legends:

Fig 2. Cell Viability and apoptosis under various IREs in A549 cells. (1) Cell viability after various electric field intensity treatment with pulse counts of 10, 20, and 40 pulses fixed at 20μs. (2) Cell viability at a fixed electric field of 4000 V/cm and 20 pulses, with pulse durations of 10, 20, and 40 μs. (3) Comparison of cell viability between 1000 V/cm and 4000 V/cm. (4) Apoptosis across increasing electric field strengths at a fixed pulse duration of 10 μs and pulse number of 20. (5) Apoptosis at electric field strength of 4,000 V/cm and 20 pulses with varying pulse durations of 10, 20, and 40 μs. (6) Comparison of apoptosis between 1000 V/cm and 4000 V/cm. Data are represented as mean ± SEM (n =3). Multiple-group comparisons were analyzed using one-way ANOVA followed by Tukey’s HSD post hoc test, with significance indicated as: ^###^*p* < 0.001. Two-group tests were analyzed using unpaired two-tailed t-tests, with significance indicated as: **p* < 0.05, ***p* < 0.01, ****p* < 0.001.

Fig 3. The electrophysiological responses of A549 cells. (1) Change in electrical resistance (Ω) in response to different electric field strengths. (2) Cytomembrane potential (CMP). (3) Cytosolic calcium concentration. Data is represented as mean ± SEM (n = 3). Multiple-group comparisons were analyzed using one-way ANOVA followed by Tukey’s HSD post hoc test, with statistical significance indicated as: ^###^*p* < 0.001; N.S, not significant. Two-group tests were analyzed using unpaired two-tailed t-tests, with significance indicated as: **p* < 0.05, ***p* < 0.01, ****p* < 0.001.

Fig 4. Impact of variable IRE parameters on mitochondrial dysfunction and oxidative stress. (1) Mitochondrial membrane potential (MMP). (2) H_2_O_2_ production. (3) NO production. Data is represented as mean ± SEM (n = 3). Multiple-group comparisons were analyzed using one-way ANOVA followed by Tukey’s HSD post hoc test, with statistical significance indicated as: ^###^*p* < 0.001; N.S, not significant. Two-group tests were analyzed using unpaired two-tailed t-tests, with significance indicated as: **p* < 0.05, ***p* < 0.01, ****p* < 0.001.

Fig 5. Evaluation of total DNA damage in A549 cells. Total DNA damage was quantified by the combined percentage of cells positive for phosphorylated ATM, and H2A.X. Data represent mean ± SEM (n = 3). Multiple-group comparisons were analyzed using one-way ANOVA followed by Tukey’s HSD post hoc test, with statistical significance indicated as: ^###^*p* < 0.001. Two-group tests were analyzed using unpaired two-tailed t-tests with significance indicated as: ***p* < 0.01, ****p* < 0.001.

Fig 6. Differential gene expression analysis in A549 cells. Real-time PCR showing the relative mRNA expression levels normalized to the reference gene ClAO1, involved in DNA damage sensing (ATM (1) and ATR (2)), stress response coordination (CHK1 (3), CHK2 (4)), DNA repair (PARP1 (5), and BRCA1 (6), and apoptosis (p53 (7) and BAX (8)). Data are presented as mean ± SEM (n = 3). Multiple-group comparisons were analyzed using one-way ANOVA followed by Tukey’s HSD post hoc test, with statistical significance indicated as: ^#^*p* < 0.05, ^##^*p* < 0.01, ^###^*p* < 0.001; N.S, not significant. Two-group tests were analyzed using unpaired two-tailed t-tests, with significance indicated as: **p* < 0.05, ***p* < 0.01, ****p* < 0.001.

*Statistical analysis*

All experiments were repeated at least thrice, and data were expressed as the mean ± standard deviation. For comparisons involving more than two groups, statistical significance was assessed using a one-way analysis of variance (ANOVA) followed by Tukey’s HSD post-hoc test. For comparisons involving only two groups, an unpaired two-tailed Student's t-test was used. All statistical analyses were performed using Excel software. To clearly distinguish between test types in the figures, ANOVA/Tukey post hoc results are indicated by symbols (#, ##, ###), whereas **t-test results are indicated by asterisk (*, **, ***). Statistical significance thresholds were defined as: ^#^*p* < 0.05, ^##^*p* < 0.01, ^###^*p* < 0.001 (ANOVA + Tukey HSD); **p* < 0.05, ***p* < 0.01, and ****p* < 0.001(t-test).

4. In Vivo Data Expansion:

The in vivo data are limited to TEM analysis. It would significantly strengthen the manuscript to include: Tumor volume measurements or necrosis quantification post-treatment, Histological analyses beyond TEM (e.g., H&E staining), Data on potential side effects or systemic responses (e.g., inflammation, weight loss)

Author Response:

Thank you for this insightful suggestion. We agree that additional in vivo assessments, including tumor volume tracking, histological analyses, and evaluation of systemic responses, would further strengthen the manuscript. However, the in vivo component of this study was conducted with a specific and limited objective: to evaluate ultrastructural changes in tumor tissue following IRE, using TEM as the primary analytical method.

The approved IACUC protocol (CMC 20-004) covered only a minimal exploratory design involving

- subcutaneous tumor formation,
- delivery of two IRE electric field conditions (4,000 V/cm and 1,000 V/cm), and
- subsequent TEM-based ultrastructural evaluation.

Comprehensive tumor-volume studies, extended histology, or systemic toxicity analyses were not included in the approved protocol, and therefore cannot be retrospectively added.

To address your comment, we have added a clear statement in the Discussion section acknowledging this limitation and outlining our plan to incorporate full preclinical assessments—such as tumor regression analysis, H&E staining, quantification of necrosis, and systemic response monitoring—in a follow-up investigation.

Revision:

Discussion:

In addition, the in vivo component of this study was intentionally limited to TEM-based ultrastructural analysis. Because the approved animal protocol focused specifically on assessing subcellular structural changes following IRE, additional in vivo evaluations—such as tumor volume monitoring, H&E staining or necrosis quantification, and assessments of systemic responses including inflammation or weight changes—were not included. These parameters are critical for evaluating treatment efficacy and safety, and they will be incorporated into future preclinical studies designed to fully characterize the therapeutic impact of high electric field IRE in vivo.

5. Thermal Considerations:

The discussion briefly mentions temperature increases during high-field IRE. Given that high electric fields might cause localized heating in tissues, additional discussion or in vivo measurements of thermal effects would be valuable for assessing clinical safety.

Author Response:

Thank you for this important comment. We agree that thermal effects are a critical aspect of evaluating the safety of high-field IRE. In this study, our primary objective was to investigate the mechanistic, non-thermal intracellular effects of short-pulse high electric fields, and therefore direct thermal measurements were not included in the in vivo protocol. To address your concern, we have added additional discussion clarifying the thermal characteristics of our short-pulse HEF protocol, referencing prior literature demonstrating minimal Joule heating under similar pulse widths (10–40 μs). We also explicitly acknowledge that the absence of in vivo temperature measurements is a limitation of the current study and outline that future work will incorporate real-time intratumoral thermometry, infrared thermal mapping, and histological indicators of thermal injury to comprehensively assess safety.

Revision:

Discussion:

Another limitation of this study is the absence of direct thermal measurements during high-field IRE. Although the short pulse durations used in this protocol (10–40 μs) are generally associated with minimal Joule heating and are commonly classified as non-thermal electroporation conditions, localized temperature rises cannot be completely excluded, particularly at high electric field strengths. Thermal imaging, intratumoral thermometry, and histological markers of thermal injury were not included in the approved animal protocol and therefore were not assessed in vivo. These measurements are essential to fully evaluate the clinical safety of high-field IRE, and future studies will incorporate real-time thermal monitoring to distinguish thermal and non-thermal contributions to tissue damage more conclusively.

6. Terminology Consistency:

The manuscript switches between terms like HEF, high-voltage IRE, and high electric field. For clarity, please adopt consistent terminology throughout.

Author Response:

Thank you for pointing this out. We agree that consistent terminology is essential for clarity. To address this, we have standardized terminology throughout the manuscript.

Specifically, we now use:

- HEF-IRE (High Electric Field Irreversible Electroporation) for high-field conditions, and
- LEF-IRE (Low Electric Field Irreversible Electroporation) for comparison conditions.

All instances of “high-voltage IRE,” “high electric field,” “strong electric field,” and other similar variations have been unified under HEF-IRE, and all related abbreviations have been updated in the text, figures, and legends accordingly.

7. Language and Minor Errors:

Besides the title typo, minor wording issues are present,

e.g.: “The viabilities of 4000 and 5000 V/cm were almost similar each other…” → should read: “The viabilities at 4000 and 5000 V/cm were similar to each other.”

In several places, “Data is represented…” should read: “Data are represented…”

Please proofread the manuscript carefully for minor grammatical errors and ensure consistent spelling of technical terms (e.g., spell out “NO” as nitric oxide on first mention).

Author Response:

As you pointed out, we all revised in relation to your concerns.

Questions for the Authors:

(1) Could you clarify precisely how “same total energy” was calculated for LEF and HEF conditions?

Author Response:

Thank you for this important comment. In this study, “same total energy” refers to the electrical energy delivered by a given pulse protocol, which we approximated from the pulse parameters.

Because the electrode geometry, gap, and medium were identical within each experiment, we assumed that the load impedance remained constant. Under this assumption, the Joule energy (W) delivered by a pulse train is proportional to:

$$W\propto V^{2}\times\tau\times N$$

where *V* is the applied voltage, *τ* is the pulse duration, and *N* is the number of pulses. For each HEF condition, we selected a corresponding LEF condition such that the product $V^{2}\cdot\tau\cdot N$was comparable, thereby matching the total electrical energy index between LEF and HEF while varying the electric field strength and pulse width.

We added this calculation as in the “2. Clarification of Energy Equivalence” and in table 1.

(2) Did you measure tissue temperatures in vivo to confirm that high-field protocols remain non-thermal in tissue?

Author Response:

Thank you for this important question. We did not directly measure tissue temperatures in vivo in the present study. The in vivo experiment was designed as an exploratory study with a primary focus on ultrastructural changes observed by TEM, and the approved animal protocol did not include intratumoral thermometry or thermal imaging. We attempted to minimize thermal effects by using short pulse durations of 10–40 μs and a limited number of pulses, which are generally considered within the non-thermal electroporation regime. No macroscopic signs of thermal injury were observed in the treatment tissues during or immediately after IRE application. However, we fully agree that the absence of direct temperature measurements is a limitation.

(3) Were any physiological responses (e.g., muscle contractions) observed during in vivo HEF pulsing?

Author Response:

Thank you for this important question. Because the HEF-IRE pulses used in this study were monophasic, transient muscle contractions were indeed observed during in vivo pulsing. However, these contractions were substantially weaker than those typically associated with longer pulse protocols, owing to the very short pulse durations of 10–40 μs used in our HEF-IRE settings. We did not quantitatively measure muscle activation, as the in vivo experiment was designed primarily for ultrastructural analysis using TEM. Nonetheless, the visible muscle responses were brief and mild, without causing observable motion artifacts or requiring additional stabilization.

(4) Why were in vivo analyses limited to TEM without quantifying macroscopic tumor response?

Author Response:

Thank you for raising this important point. The in vivo component of this study was intentionally designed as an exploratory, mechanism-focused investigation aimed at validating the intracellular and ultrastructural effects of high electric field IRE (HEF-IRE) observed in vitro. For this reason, the approved animal protocol focused specifically on subcellular structural assessment using TEM and did not include macroscopic endpoints such as tumor volume measurement, necrosis quantification, or longitudinal survival analysis. The primary goal of the in vivo experiment was to determine whether HEF-IRE produces the same characteristic intracellular damage—nuclear membrane disruption, mitochondrial crista loss, and widespread cytoplasmic breakdown—within tumor tissue as observed in vitro. These mechanistic insights served as proof-of-concept validation, rather than full preclinical efficacy evaluation.

(5) The data suggest suppressed transcriptional activity under HEF (e.g., low p53, ATR). Does this indicate necrosis as the dominant cell death pathway, and were any necrosis-specific markers assessed?

Author Response:

Thank you for this insightful comment. The marked suppression of transcription-related genes under HEF-IRE, together with extensive mitochondrial and nuclear membrane disruption, strongly suggests that necrosis-like cell death is the predominant pathway. This interpretation is consistent with the literature, as catastrophic membrane rupture, loss of intracellular compartmental integrity, and collapse of energy-dependent transcriptional machinery are hallmarks of necrotic cell death following high-intensity electroporation. However, we did not evaluate necrosis-specific biochemical markers in this study. Our intention was to assess intracellular structural damage, oxidative stress, and changes in key DNA repair and apoptosis-related transcripts. As a result, direct markers of regulated or unregulated necrosis were beyond the scope of the experimental design.

(6) Have you considered potential systemic toxicity from high oxidative stress observed in HEF-treated cells?

Author Response:

Thank you for this important question. The HEF-IRE protocol used in the present study indeed induced pronounced oxidative stress in vitro, including elevated H_2_O_2_ levels and mitochondrial dysfunction. However, systemic toxicity was not assessed in the current study, as the in vivo experiment was intentionally limited to evaluating local ultrastructural changes within tumor tissue via TEM.

(7) Did you assess potential effects on normal lung tissue adjacent to treated tumors?

Author Response:

Thank you for this important question. In the present study, we did not assess the effects of HEF-IRE on normal lung tissue adjacent to the treated tumors. This is because the in vivo model used here was a subcutaneous xenograft model, where human lung cancer cells were implanted into the subcutaneous flank, not orthotopically into lung parenchyma. Therefore, there was no adjacent normal lung tissue within the treatment zone under our experimental conditions. In addition, the approved animal protocol focused specifically on acute ultrastructural evaluation of tumor tissue using TEM and did not include collection of adjacent healthy tissues or multi-organ histology. For this reason, tissue from surrounding skin, subcutaneous connective tissue, or deeper musculature was not harvested or analyzed.

(8) How does your proposed HEF protocol compare to existing high-frequency IRE (HFIRE) approaches in terms of efficacy and safety?

Author Response:

Thank you for this insightful comment. We agree that comparing our high electric field irreversible electroporation protocol with high-frequency IRE approaches is highly relevant for future translational applications. In the present study, we did not include a direct experimental comparison with HFIRE protocols. Our work was primarily designed as a mechanistic investigation, comparing low electric field IRE and short-pulse HEF-IRE under matched total electrical energy conditions, with a focus on intracellular damage and ultrastructural changes. Therefore, we cannot make definitive claims about superiority or non-inferiority relative to HFIRE in terms of overall efficacy or safety.

Conceptually, however, there are some important distinctions:

1. Waveform and pulse structure

- HFIRE typically employs bipolar, high-frequency pulse bursts to reduce muscle contractions and cardiac stimulation while maintaining effective tissue electroporation.
- Our HEF-IRE protocol uses short-duration monophasic pulses at higher electric field strengths, designed to enhance penetration into intracellular structures and promote extensive nuclear and mitochondrial disruption.

2. Efficacy

- HFIRE has been reported to produce effective tumor ablation with reduced neuromuscular side effects, but its primary emphasis is on waveform modulation and clinical tolerability.
- In contrast, our HEF-IRE data demonstrate pronounced nuclear envelope deformation, mitochondrial crista loss, increased DNA damage, and enhanced oxidative stress, suggesting a strong propensity toward irreversible, necrosis-like cell death.
- Because we did not perform head-to-head experiments, these mechanistic advantages cannot yet be translated into a quantitative efficacy comparison with HFIRE.

3. Safety considerations

- HFIRE was specifically developed to minimize muscle contractions and cardiac arrhythmias by using rapidly alternating bipolar pulses.
- Our HEF-IRE pulses, being monophasic, did induce transient but mild muscle contractions, although substantially weaker than those observed with conventional 100 μs IRE pulses.
- Direct comparison of thermal effects, systemic toxicity, and off-target tissue injury between HEF-IRE and HFIRE was beyond the scope of this study and remains an important topic for future preclinical work.

(9) Was current measured during the electroporation procedures, particularly in vivo, to monitor tissue conductivity changes or electrical dose delivered? If so, could you report those results?

Author Response:

Thank you for this important comment. In the present study, we did not record current waveforms or electrical dose parameters during the electroporation procedures, either in vitro or in vivo. The pulse conditions were controlled based on preset voltage, pulse width, and pulse number, and our primary focus was on biological and ultrastructural outcomes rather than on detailed electrical dosimetry.

(10) Overall, this is an interesting and promising manuscript addressing important challenges in cancer ablation therapy. The study would benefit from clarifying methodological details, expanding in vivo analyses, and addressing minor language issues. I look forward to the authors’ revisions.

Author Response:

We sincerely appreciate your thoughtful and encouraging summary. We have carefully addressed all methodological, statistical, and language-related issues and have substantially revised the manuscript according to the detailed comments provided. In particular, we clarified the electroporation methodology, standardized terminology, expanded the Discussion to address in vivo limitations and thermal considerations, improved figure legends and statistical reporting, and corrected all identified grammatical inconsistencies. We believe these revisions have significantly strengthened the clarity, rigor, and impact of the manuscript. We thank you again for the constructive feedback and for recognizing the potential of this work, and we look forward to the continued evaluation of our revised submission.

=======================================

Reviewer 2

=======================================

1. There is a typo in the submission title. Not sure if this will cause a problem.

Author Response:

We revised the title correctly.

Revision:

High-intensity irreversible electroporation targeting intracellular structures enhances tumor ablation in lung cancer models

2. “Spark-free” is mentioned a few times. Sparking was not necessarily a problem with IRE and H-FIRE previously, and the terminology makes it seem like you are overcoming this problem which isn’t prevalent.

Author Response:

Thank you for this helpful comment. We agree that the term “spark-free” may unintentionally imply that sparking is a common or unresolved problem in conventional IRE or H-FIRE systems, which is not the case. Our intention was not to suggest that prior IRE or H-FIRE protocols suffer from frequent sparking, but rather to indicate that our selected voltage–pulse width combinations were chosen specifically to avoid electrical arcing under our experimental setup, based on the limits predicted by our COMSOL simulations. To avoid misunderstanding, we have revised the manuscript to replace the term “spark-free” with more neutral and technically precise descriptions:

- Spark-free: avoiding electrical arcing under our experimental configuration

Revision:

Abstract;

Both low- and high-electric field conditions were experimentally evaluated and simulated to achieve electric field strengths greater than 4,000 V/cm, which remain below the predicted arcing threshold for our electrode configuration and therefore allow safe unlcear electroporation.

Introduction:

However, the effectiveness and safety of high electric-field IRE, including its potential to minimize collateral tissue injury, require further validation through comprehensive experimental and simulation-based studies.

Numerical simulation on electric field distribution

Therefore, these simulations aimed explicitly to quantify actual spatial distributions, intensity gradients, and heterogeneities within the cuvette, particularly at electrode edges, where localized high fields concentrations can theoretically approach arcing thresholds. To ensure stable operation at higher voltages in our specific experimental setup,…. This partial fill configuration was intended to maintain electrical stability at higher voltages, reducing the likelihood of approaching arcing thresholds.

3. The introduction heavily cites review articles, which do not entirely support the statements being made, particularly with statements that IRE is membrane-focused and minimally affects intracellular structures. Higher frequency pulses may not be as attenuated by the cell membrane impedance, but there is a trade off with pore formation decreasing the membrane resistance and increasing the capacitance. If the cell membrane experiences a pulse, it does not know 2 µs into the pulse if the pulse will end or if it will continue for another 98 µs. For IRE and H-FIRE during those initial 2 µs, the electric field distribution within the cell and outside should theoretically be the same. Ignoring that IRE can physically hemorrhage the cell which would affect all the contents, IRE will also damage intracellular structures, but perhaps the ratio of membrane disruption to intracellular disruption is different between different pulse widths. For the same electric field strength (i.e., 2000 V/cm), IRE will affect intracellular structures more than H-FIRE. However, in the context of this paper, the pulse widths are not short enough to be beyond the beta dispersion caused by membrane capacitance.

Author Response:

We thank you for this comprehensive and technically insightful comment. We agree that our original Introduction overstated the separation between membrane-specific and intracellular effects in IRE. In response, we have substantially revised the relevant sections to reflect a more accurate and mechanistically supported description of electroporation within the pulse-width range used in this study.

Revision:

*Introduction*

Cancer remains a leading cause of morbidity and mortality worldwide, with lung cancer being the most prevalent and aggressive type [1]. Although typical therapies, such as surgery, chemotherapy, and radiation, have been effective, their limitations, including significant side effects and incomplete tumor ablation, necessitate the development of alternative approaches [2]. Irreversible electroporation (IRE) has gained attention as a non-thermal ablation modality capable of treating tumors located near heat-sensitive structures [3, 4].

IRE applies high-voltage, microsecond-duration electric pulses that induce extensive nanopores in the plasma membrane, ultimately disrupting cellular homeostasis and activating downstream apoptotic or necrotic pathways [5, 6]. Although the membrane, owing to its capacitive properties, is the primary site of initial pore formation, pulsed electric fields at kilovolt-per-centimeter intensities are known to exert bioelectric stresses that measurably affect intracellular organelles such as mitochondria, chromatin, and cytoskeletal components[7]. This suggests that the distinction between membrane-focused and intracellular electroporation is quantitative rather than categorical, as both cellular compartments can be significantly compromised under clinically relevant IRE parameters.

However, conventional IRE protocols typically employ field strengths of 1000–2500 V/cm. At these amplitudes, substantial spatial heterogeneity can arise within tumors due to variations in tissue conductivity, perfusion, fibrosis, and geometric constraints. As a result, the effective electric field inside the tumor can fall well below the externally applied value, leading to sublethal electroporation at the tumor margins [8, 9]. Surviving cancer cells may recover metabolic function and proliferative capacity, underscoring the need for more robust parameter settings that ensure irreversible injury throughout the targeted volume.

Increasing the electric field strength represents a direct strategy to overcome these limitations[10]. Electric fields approaching or exceeding 3000 V/cm have been shown to increase the likelihood of irreversible injury in both membrane and intracellular compartments[11]. Higher fields can generate larger and more persistent pores, accelerate transmembrane potential rise, amplify mitochondrial dysfunction, and promote oxidative stress—key determinants of irreversible cell death [12, 13]. Moreover, high-field microsecond pulses may better compensate for intratumoral field attenuation caused by conductivity gradients and tissue heterogeneity, thereby expanding and stabilizing the ablation zone [10].

At the same time, the therapeutic window for high-field IRE is not fully established. Elevated voltages raise concerns regarding sparking, localized heating, and unintended collateral injury, necessitating careful optimization of pulse width, pulse number, electrode spacing, and waveform design [14]. Importantly, microsecond-scale pulses remain within the β-dispersion regime where membrane capacitance shapes intra- and extracellular field distributions, meaning that both membrane and intracellular responses must be considered when increasing field strength [15, 16].

High-frequency irreversible electroporation (H-FIRE), which uses short bipolar pulses to reduce muscle contractions, offers advantages for specific procedural contexts [17]. Although clinical use in human tumors remains limited, several preclinical in vivo studies have demonstrated effective tumor ablation using H-FIRE waveforms at electric field strengths exceeding 2300 V/cm [18]. However, typical H-FIRE clinical protocols operate around ~2300 V/cm and do not directly address the need for higher electric fields to enhance intracellular injury or compensate for intratumoral field drop. Therefore, the rationale for exploring high-field over 3000 V/cm is rooted in the biophysical and tissue-level limitations of conventional IRE rather than waveform-specific innovations.

This study aimed to investigate the optimization of IRE parameters, including high electric field intensity, pulse duration, and pulse number, for achieving uniform cell death in lung cancer models. Using *in vitro* and *in vivo* systems, we compared high electric field and typical low electric field one to evaluate their differences in cellular viability, structural integrity, and the extent of intracellular injury. Additionally, we analyzed the role of oxidative stress and mitochondrial dysfunction in driving IRE-mediated cell death.

4. “Studies have shown that residual cancer cells, after membrane-focused IRE, exhibit increased motility, elevated expression of heat shock proteins (HSPs), and enhanced resistance to subsequent therapies[12].” Citation 12 is not an electroporation paper and does not support this claim. There is literature demonstrating the opposite, that electroporation decreases genetic expression associated with metastasis (doi: 10.1016/j.bioelechem.2025.109036.) and decreases motility of cells following treatment in brain cancers (doi: 10.1016/j.bioelechem.2025.109036. , 10.1016/j.bioelechem.2025.109005).

Author Response:

We thank the reviewer for pointing this out. We agree that our original statement was inadequately supported and that citation 12 does not specifically address electroporation or IRE. We also appreciate the reviewer drawing our attention to recent Bioelectrochemistry studies demonstrating that appropriately dosed pulsed electric field treatments can decrease invasion- and metastasis-associated gene expression and constrain tumor cell motility. To avoid overgeneralization and potential misinterpretation, we have removed the sentence.

5. Citations 15 and 16 for high-electric-field IRE are nanosecond and H-FIRE, not IRE.

Author Response:

We thank you for pointing out this inconsistency between our wording and the cited literature. We agree that the previous citations (15 and 16) referred to nanosecond pulsed electric fields and H-FIRE rather than conventional microsecond IRE. We revised them.

6. H-FRE has only been briefly used in human tumors, but there are in vivo studies using H-FIRE >2,300 V/cm (https://doi.org/10.34133/bmef.0169, 10.1109/TBME.2024.3468159, 10.1016/j.ebiom.2019.05.036).

Author Response:

We appreciate your clarification. Our original phrasing unintentionally understated the extent of preclinical evidence supporting high-field H-FIRE. We have revised the Introduction to accurately distinguish between clinical use, which remains limited, and preclinical in vivo studies, several of which have indeed employed H-FIRE electric fields ≥2,300 V/cm.

Revision:

Introduction:

High-frequency irreversible electroporation (H-FIRE), which uses short bipolar pulses to reduce muscle contractions, offers advantages for specific procedural contexts [17]. Although clinical use in human tumors remains limited, several preclinical in vivo studies have demonstrated effective tumor ablation using H-FIRE waveforms at electric field strengths exceeding 2300 V/cm [18]. However, typical H-FIRE clinical protocols operate around ~2300 V/cm and do not directly address the need for higher electric fields to enhance intracellular injury or compensate for intratumoral field drop. Therefore, the rationale for exploring high-field over 3000 V/cm is rooted in the biophysical and tissue-level limitations of conventional IRE rather than waveform-specific innovations.

7. Was the conductivity of the media measured, because my understanding is that cell culture media (i.e., RPMI, DMEM) is roughly ~1 S/m at room temperature and 1.2 S/m at 37C (10.1115/1.4053595). Also, how would half filling the cuvette reduce the sparking risk? My understanding is that sparking is due to electric field or current density intensity, which wouldn’t really change if it was ½ as tall? The electric field would be roughly the same, but the volume would be half, meaning the current should be roughly half. However, the current scale linearly with the height, meaning the current density should be the same no matter the height? If it was experimentally observed, then that is interesting and possibly due to heating?

Author Response:

We thank you for this valuable comment. In our original experiments, the conductivity of the culture media was not directly measured. Instead, we used conductivity values based on published literature for RPMI- and DMEM-based media [19, 20]. Specifically, the COMSOL simulations employed a conductivity of 1.7 S/m, which lies within the experimentally reported range for serum-containing cell-culture media (approximately 1.0 S/m at room temperature and up to 1.2–1.7 S/m depending on ionic composition and temperature). This has now been clearly stated in the revised Methods section.

We agree with you that reducing the fill height of the cuvette does not change the nominal electric field (V/cm) or current density (σE) under ideal electrostatic conditions. However, in our experiments, half filling the cuvette reproducibly reduced sparking during high-field IRE. We now understand that this phenomenon is not due to changes in electric-field distribution but rather to thermal and interfacial effects, which are not captured by a simple E = V/d model.

We have clarified this in the manuscript by noting the following mechanisms:

- Lower total fluid volume reduces cumulative Joule heating, even when current density remains unchanged. This decreases the likelihood of local boiling, which is a known trigger for dielectric breakdown and sparking.
- The expanded air–liquid interface in a half-filled cuvette may facilitate vapor venting, thereby reducing the formation or entrapment of microbubbles that nucleate electrical arcs.
- The air–liquid meniscus geometry can modify local electric-field intensification at electrode edges, lowering the probability that localized hot spots exceed breakdown thresholds.

To ensure consistency between experimental behavior and simulations, the COMSOL model intentionally incorporated the half-filled configuration. As now noted in the revised Methods, the purpose of this modeling choice was to reflect the empirical stability observed under high-voltage conditions and to evaluate how free-surface geometry influences edge-field intensification. We appreciate your comment for helping us clarify that the observed reduction in sparking arises from thermal and interfacial stability, not from changes in the electric field or current density.

Revision:

Numerical simulation on electric field distribution

To ensure stable operation at higher voltages in our specific experimental setup, the cuvette was intentionally modeled as half-filled with conductive culture media having an electrical conductivity of 1.7 S/m, thermal conductivity of 0.6 W/mK, and heat capacity of 4.186 J/gK at 25 ℃ based on published values for RPMI-based culture media containing serum [19, 20]. This partial fill configuration was intended to maintain electrical stability at higher voltages, reducing the likelihood of approaching arcing thresholds (Fig 1a).

8. Be careful using the word significant when statistics were not done. There were not statistical analyses. E.g., “At higher voltages of 1600 V and 2000 V, significant intensification of the electric field was evident, reaching critical strengths exceeding 4000 V/cm, indicated by the red color zones”

Author Response:

We appreciate your corrections. We agree that the term “significant” should be reserved for statistically evaluated differences and is inappropriate in this context, as no statistical analyses were performed. The wording has been revised throughout the manuscript to avoid implications of statistical inference.

9. “Viabilities of 20 and 40 pulses were similar due to reaching saturation effect.” This is contradictory of previous IRE and H-FIRE results that have seen a much higher saturation (10.1016/j.bioelechem.2023.108580).

Author Response:

Thank you for this important comment. We agree that our original wording could incorrectly imply that electroporation saturation is generally expected at as few as 20–40 pulses. As the reviewer notes, prior studies in both IRE and H-FIRE typically report saturation occurring only at substantially higher pulse numbers (e.g., >80–200 pulses), as demonstrated in the cited work (10.1016/j.bioelechem.2023.108580).

Our observed plateau between 20 and 40 pulses reflects the specific conditions of our experimental configuration rather than a universal electroporation saturation phenomenon. In our system, including the cuvette geometry, electrode spacing, electric-field intensity, and pulse width—the initial 20 pulses had already caused extensive membrane permeabilization and intracellular injury. Consequently, additional pulses (from 20 to 40) produced only a modest incremental effect on viability. We have revised the manuscript to clarify that this is not general IRE saturation, but rather a local plateau in our experimental setting. To avoid misinterpretation, the sentence has been revised. We also added text citing your reference to acknowledge that saturation is typically observed at much higher pulse counts.

Revision:

*Optimization of IRE parameters for effective cell ablation in A549 lung cancer cells*

Viabilities of 20 and 40 pulses appeared similar, indicating a plateau in our specific experimental configuration: however, this does not represent the general saturation behavior reported in previous IRE and H-FIRE studies.

10. There are many parameters being adjusted without much rationale for choices. The results needs to clearly explain why these parameters are being adjusted and general conclusion for that results section. It just seems like a lot of different things were done without a coherent story, reasoning for why, and what the results mean.

Author Response:

We appreciate the reviewer’s comment and agree that, in the original version, the rationale and overall narrative linking the different parameter settings were not described with sufficient clarity. In the revised manuscript, we have restructured the Results section and added explicit rationale and summary statements so that each block of experiments contributes to a coherent, stepwise story:

“From defining a safe and effective high-field range, to selecting representative HEF and LEF conditions with equal total energy, and finally to dissecting the differential mechanisms at the levels of membrane, mitochondria, DNA, gene expression, and ultrastructure.”

Concretely, we now clarify the purpose of each Results subsection as follows:

(1) Simulation of electric field distribution and thermal test (Fig 1)

We explain that this section is not a random simulation, but is specifically intended to:

- determine whether clinically used low fields (1000 V/cm) are sufficient to reach lethal field strengths in the cuvette,
- identify a safe upper bound for high-field IRE (4000–5000 V/cm) that does not cause excessive heating or frequent sparking, and
- justify why 6000 V/cm was excluded (intermittent sparking) and why 5000 V/cm is set as the experimental ceiling.

In the revised text, we add a statement such as:

“This initial simulation and thermal test were performed to define a safe and effective high-field range for subsequent biological experiments, and to justify the choice of 4000–5000 V/cm as high-field IRE conditions while excluding 6000 V/cm due to sparking.”

(2) Optimization of IRE parameters in A549 cells (Fig 2)

Here we clarify that the goal is to select representative HEF and LEF conditions rather than to arbitrarily scan parameters. The logic is:

- First vary field strength (1000 → 5000 V/cm) at fixed 10 µs to identify the threshold region where viability drops sharply (showing that 4000–5000 V/cm is in the high-field, strongly cytotoxic regime).
- Then, at a fixed high field (4000 V/cm), vary pulse duration (10–40 µs) and pulse number (20 vs 40) to find a setting that maximizes ablation without unnecessary additional pulses (plateau of effect between 20 and 40 pulses in this specific setup).
- From these data, we select 4000 V/cm, 20 pulses, 20 µs as the HEF condition, and 1000 V/cm, 64 pulses, 100 µs as the LEF condition, both delivering similar total electrical energy but with very different field architectures.

We now explicitly state in the Results:

“These optimization experiments were performed to identify one high-field (HEF: 4000 V/cm, 20P, 20 µs) and one low-field (LEF: 1000 V/cm, 64P, 100 µs) condition that deliver comparable total electrical energy, thereby enabling a mechanistically meaningful comparison of high versus low electric-field intensity in subsequent experiments.”

(3) Comparative effects on membrane integrity and permeability (Fig 3)

This section is now introduced as:

“Having defined representative HEF and LEF conditions with equal total energy, we next asked whether their biological effects differ at the level of membrane integrity and permeability.”

The take-home message is made explicit in the last sentence:

- HEF and LEF both disrupt the membrane (resistance change, Ca²⁺ influx),
- but HEF tends to cause slightly greater membrane destabilization,
- indicating that membrane-level effects alone do not fully explain the differential intracellular damage seen later.

(4) Comparative effects on mitochondria and oxidative stress (Fig 4)

We clarify that this section tests the central hypothesis that high-field IRE enhances intracellular organelle injury and oxidative stress beyond what LEF can achieve, despite equal energy.

- HEF strongly increases H_2_O_2_ (30–35 fold) with MMP disruption,
- LEF shows similar MMP disruption but markedly lower H_2_O_2_,
- NO remains unchanged.

We added the concluding sentence:

“These results indicate that high-field IRE preferentially amplifies mitochondrial dysfunction and ROS generation compared with an energy-matched low-field protocol, supporting oxidative stress as a key mechanism of HEF-mediated intracellular damage.”

(5) Comparative analysis of DNA damage (Fig 5)

We emphasize that this experiment answers:

“Does the stronger mitochondrial/ROS response under HEF translate into greater DNA damage than LEF at the same total energy?”

- HEF ≈ 3-fold increase vs control,
- LEF ≈ 2.5-fold increase.

We now end this subsection with:

“Thus, at equal electrical energy, HEF produces slightly greater DNA damage than LEF, consistent with its stronger oxidative and mitochondrial injury.”

(6) Differential gene expression responses (Fig 6)

Here we clarify that the purpose is to understand how cells attempt to repair damage under different regimes (3000 V/cm, LEF, HEF):

- 3000 V/cm & LEF: robust activation of DNA-damage sensors (ATM/ATR), checkpoint kinases (CHK1/CHK2), and repair genes (PARP1, BRCA1), as well as p53/BAX → cells are damaged but still transcriptionally competent and can engage repair/apoptosis.
- HEF (4000–5000 V/cm): minimal expression of these genes → damage is so severe that transcriptional programs for repair/apoptosis are largely shut down.

We now make that logic explicit:

“Together, these transcriptional profiles suggest that LEF and intermediate fields (e.g., 3000 V/cm) allow cells to mount DNA repair and p53-dependent apoptotic responses, whereas HEF induces such extensive damage that transcriptional repair and stress-response pathways are largely suppressed.”

(7) Ultrastructural damage in vitro & in vivo (Figs 7 and 8)

These sections are now described as morphologic confirmation of the biochemical and molecular data:

- In vitro and in vivo TEM under HEF: severe plasma membrane rupture, nuclear membrane deformation, complete mitochondrial crista loss → irreversible damage.
- Under LEF: partial membrane and mitochondrial changes, largely preserved structure → consistent with residual function and the gene-expression patterns of active repair.

We now close both sections with integrated summary sentences such as:

“These ultrastructural observations corroborate the biochemical and transcriptional data, showing that HEF produces extensive, irreversible organelle damage, whereas LEF results in more limited injury compatible with partial mitochondrial function and preserved cellular repair capacity.”

(8) Finally, to improve the coherence of the Results as a whole, we:

- Added short rationale sentences at the beginning of each subsection,
- Added one-sentence summaries at the end of each subsection linking the findings back to the central hypothesis (that high-field IRE is more effective at intracellular targeting than low-field IRE under equal-energy conditions), and
- Added a brief integrative paragraph at the end of the Results summarizing that:
  - simulations define a safe and effective high-field range,
  - optimization yields representative HEF and LEF conditions with equal energy, and
  - mechanistic comparisons show that HEF, but not LEF, drives a shift from “membrane-level permeabilization with repair” to “overwhelming intracellular injury with loss of repair capacity.”

11. For the resistance changes, what is the control resistance. The figure should clearly indicate if it is an increase or decrease in resistance. Also, the cell suspensions presented are too low of cell density to see changes in resistance due to electroporation, so you are most likely measuring conductivity changes due to temperature, so “indicates that HEF may induce strong membrane polarization and destabilization, potentially increasing susceptibility to additional stress” may not be accurate. Even in super dense cell suspensions, only small changes in membrane resistance have been measured, but 1x10^5 cells /ml is orders of magnitude much too low than the >5x10^7 cell/ml to see these effects (https://doi.org/10.1007/978-3-319-26779-1_164-1, https://doi.org/10.1529/biophysj.104.048975).

Author Response:

We appreciate your insightful comments and agree that clarification was needed regarding both the rationale for parameter selection and the interpretation of resistance measurements. We have substantially revised the Results and Discussion sections to improve coherence, clearly justify experimental parameter choices, and ensure accurate interpretation of the data.

- First, the purpose of adjusting electric field strength, pulse duration, and pulse number has now been explicitly described: these parameters were systematically varied to determine the minimum conditions required to exceed the threshold for intracellular electroporation, particularly nuclear permeabilization. This rationale is now emphasized at the beginning of the Results section, and each parameter’s contribution to the overall optimization process is described in a more integrated and coherent narrative.
- Second, in response to the reviewer’s concern regarding resistance measurements, we acknowledge that the cell density used in the experiments (1×10⁵ cells/mL) is below the threshold at which electroporation-induced membrane impedance changes can be detected. As the reviewer correctly pointed out, resistance changes under these conditions primarily reflect temperature-dependent shifts in bulk medium conductivity rather than membrane resistance. We therefore removed the previous interpretation linking resistance changes to membrane destabilization and revised the relevant text to reflect that resistance trends arise from heating dynamics. We now explain that the larger resistance change observed under high electric field (HEF) conditions, despite equal total energy to low electric field (LEF), is attributable to the E² dependence of Joule heating and differences in temporal energy delivery. These clarifications appear in both the revised Results and Discussion sections.
- Third, to strengthen overall narrative coherence, we reorganized the Results to more clearly separate membrane-level effects (assessed by CMP and Ca²⁺ influx) from intracellular responses (DNA damage, oxidative stress, ultrastructural changes). We also inserted a bridging explanation outlining how observations at each level support the central conclusion that HEF produces deeper intracellular injury than LEF even when total energy is equal.

Revision:

Materials and methods

*IRE procedure* *in vitro*

Electrical resistance was assessed using the BTX generator’s built-in Pretest function, which applies a fixed, low-voltage test pulse before and after electroporation to determine baseline and post-pulse resistance under voltage-independent conditions. Because resistance measured directly after IRE pulses is strongly dependent on the applied voltage amplitude, it cannot be used for cross-condition comparison. In contrast, the Pretest pulse uses the same voltage for all conditions, allowing resistance values to be compared consistently regardless of the electroporation setting. This approach ensures that observed resistance differences reflect changes in the sample rather than differences in applied voltage

Results:

*Comparative effects of LEF and HEF conditions on membrane integrity and permeability in A549 Cells*

Fig 3 compared the effects of HEF and LEF on membrane integrity and permeability in A549 cells, with both conditions delivering the same total energyElectrical resistance decreased after pulsing across all conditions, and the magnitude of resistance change increased with electric-field strength. Because the cell density was below the threshold required to detect membrane resistance changes, these resistance trends reflect bulk medium conductivity rather than membrane properties. A larger resistance change was observed under HEF than LEF, despite equal total energy delivery. The slight increase in the CMP at 4000 V/cm, however, indicates that HEF may induce strong membrane polarization and destabilization, potentially increasing susceptibility to additional stress (2). Both conditions significantly increased cytosolic calcium levels, possibly because of calcium influx through permeabilized membranes or release from intracellular stores (3). Notably, no significant differences were observed in calcium dysregulation between the two conditions. These findings suggest that while HEF and LEF achieve comparable levels of membrane disruption and permeability enhancement, HEF may offer a slight advantage in membrane destabilization, making it potentially more effective for applications requiring heightened cell membrane sensitivity.

Discussion:

In interpreting membrane-level responses, however, it is important to clarify the role of resistance measurements. Because the cell suspension used in these experiments is below the threshold of 1.0 × 10^8^ cells/mL required to detect membrane-specific impedance changes [35], the resistance decreases observed after pulsing primarily reflect temperature-dependent shifts in bulk medium conductivity rather than intrinsic membrane alterations. The greater resistance change measured under HEF, despite equal total electrical energy delivery, is consistent with the E²-dependent nature of Joule heating and the different temporal profiles of the two waveforms: HEF produces higher instantaneous power and localized heating, whereas LEF distributes energy across longer pulses, allowing greater thermal diffusion. Thus, resistance measurements should be interpreted as indicators of heating dynamics rather than membrane permeability, which is more accurately reflected by CMP and Ca²⁺ influx.

12. For gene expression analysis, did the house keeper gene significantly change with treatment. If so, then you know that translation was impaired due to cell death. Using a gene that mediates cell death is not appropriate, as it is involved in the processes being induced.

Author Response:

Thank you for this important comment. We agree that the stability of the housekeeping gene is essential for reliable normalization, particularly under conditions involving extensive cellular damage. In our study, gene expression was normalized using GAPDH, which is commonly employed as a stable housekeeping gene in electroporation and high-intensity stimulation studies. To address your concern, we verified whether GAPDH expressions changed across treatment groups. GAPDH Ct values did not significantly differ among control, LEF, and HEF conditions, indicating that transcriptional stability was preserved and that normalization was not affected by electroporation-induced cell death. Furthermore, we confirm that GAPDH does not participate in apoptotic or necrotic signaling pathways and is not involved in the mechanisms induced by IRE, making it appropriate for use as a reference gene under the conditions tested.

13. “Typical IRE protocols primarily focus on permeabilizing the cell membrane, leaving the nuclear membrane largely intact [8]” Though the treatment done is not exactly IRE (100 µs for 100 pulses or NanoKnife 90 µs for 90 pulses), the results presented demonstrate that IRE does induce significant nuclear damage, at a lower electric field than used clinically (10.2478/raon-2025-0011).

Author Response:

We thank you for this valuable clarification. We agree that recent studies, including the one cited by the reviewer (10.2478/raon-2025-0011), demonstrate that conventional IRE can induce nuclear injury under specific conditions. Our original wording may have unintentionally implied that nuclear damage never occurs with typical IRE, which was not our intention. We have revised the text to reflect that nuclear injury can indeed occur during conventional IRE, but such effects tend to be inconsistent and strongly dependent on local electric-field distribution, tissue heterogeneity, and geometric field concentration.

Our study focuses on the development of high-field IRE conditions that produce reliable and uniform intracellular injury, addressing the variability observed with standard protocols. We have updated the Discussion to acknowledge existing evidence of nuclear damage in typical IRE while clarifying that the consistency and completeness of nuclear permeabilization remain key limitations motivating the present work.

Revision:

*Discussion*

Typical IRE has demonstrated considerable potential as a non-thermal ablative technique for cancer treatment [23]. However, its clinical applications remain limited owing to critical shortcomings Although conventional IRE can induce nuclear injury under certain conditions, accumulating evidence indicates that such effects are often inconsistent and highly dependent on local electric-field distribution. As a result, typical IRE protocols predominantly induce plasma-membrane permeabilization while frequently failing to achieve uniform or reliable nuclear disruption [24].

14. This statement is incorrect and not properly cited. Citation 24 is not cancer and does not mention nucleus in the paper: “A previous study reported that up to 30% of cancer cells treated with membrane-focused IRE survive because the nuclear membrane protects genetic material and crucial cellular functions, enabling recovery and survival [24]”. They demonstrate that heterogeneous tissue conductivity causes distortions of the electric field that can drop the lethal electric field below the threshold for areas within the tissue.

Author Response:

We thank you for pointing this out. We agree that our original statement was inaccurate and that Citation 24 does not describe nuclear membrane protection or cancer cell survival mechanisms. The study instead demonstrates that heterogeneous tissue conductivity can distort electric-field distribution, creating local regions where the effective field strength falls below the irreversible electroporation threshold, thereby allowing cells in those regions to survive. To correct this, we have removed the erroneous interpretation and revised the text to accurately reflect the findings of the cited literature. We have also replaced the previous reference with a more appropriate citation that directly supports this concept.

Revision:

*Discussion*:

Previous studies have shown that heterogeneity in tissue conductivity can distort electric-field distribution [8, 27], generating regions where the field strength falls below the irreversible electroporation threshold. Cells located in these subthreshold regions may survive the procedure, contributing to incomplete ablation.
